# The burden of clinically significant symptoms of common and severe mental disorders among adults in Vietnam: a population-based cross-sectional survey

**DOI:** 10.1186/s12889-019-7513-7

**Published:** 2019-08-27

**Authors:** Trang Nguyen, Tuan Tran, Ha Tran, Thach Tran, Jane Fisher

**Affiliations:** 1 School of Public Health and Preventive Medicine, Monash University, 553 St Kilda Road, Melbourne, Victoria 3004 Australia; 2 Research and Training Centre for Community Development (RTCCD), No 39, lane 255, Vong street, Hanoi, Vietnam

**Keywords:** Common and severe mental disorders, Risk factors, Vietnam, Adults

## Abstract

**Background:**

Vietnam has limited evidence about the burden of common and severe mental disorders among adults to inform policy. The aim of this paper was to estimate the prevalence of common and severe mental disorders among adults and factors associated with them in Vietnam.

**Methods:**

We conducted a cross-sectional household survey among people aged at least 16 years in Thanh Hoa and Ben Tre provinces which are nationally representative of the North and the South of Vietnam. The World Health Organization Self-Reporting Questionnaire 24 was used to screen for clinically significant symptoms of common and severe mental disorders at the individual level. Household characteristics were obtained in face-to-face interviews with the household heads. A multilevel mixed-effects logistic regression model was used to identify associated factors of the common and severe mental disorders.

**Results:**

Among 611 households which included 1528 adults, the point prevalence of clinically significant symptoms was 14.4% for common mental disorders and 8.2% for severe mental disorders after weighting by age groups. Common mental disorders were associated with social factors including lived in a Northern rather than a Southern province, disadvantaged household economic status, in which a family member(s) misused alcohol, the family lacking links to social organisations able to provide instrumental support, and the individual not having completed primary school. Severe mental disorders had fewer associations with social factors compared to common mental disorders, but were associated with living in the Northern province, disadvantaged household economic status, family violence and being older than 50 years.

**Conclusions:**

The prevalence of clinically significant symptoms of common and severe mental disorders among adults in Vietnam was higher than in high income countries and had a strong association with household characteristics. The result indicates that a community-based approach to reduce household risk factors and to provide instrumental support might be an effective strategy to alleviate the burden of mental health problems in Vietnam.

## Background

Mental disorders in low and middle-income countries (LMICs) are becoming more widely recognized due to its burdens and associating factors [1]. Mental disorders contributed to more than 40% of years lived with disability (YLDs) [2, 3] and nearly 15% of disability-adjusted life years (DALYs) globally [4]. The term common mental disorders (CMDs) is the widely-accepted descriptor for non-psychotic anxiety, depressive, adjustment and somatic disorders which are potentially detectable in primary care [5]. Severe mental disorders (SMDs) include disorders characterised by psychotic symptoms such as schizophrenia and bipolar affective disorder [6]. The World Health Organization (WHO) classifies psychotic symptoms as abnormalities in thinking or disorganized behaviour: delusions, hallucinations, neglect of self-care, inability to complete usual responsibilities and either lack of volition or the excessive drive revealed in manic states [7].

Depressive disorders was the third leading cause of disability in 2017 [3]. It is estimated that 80% of the people experiencing both these conditions live in LMICs [8]. Steel et al’s (2014) recent systematic review of 174 population-based studies from 63 countries, reported a pooled 12-month period prevalence of anxiety and depression of 20%. The review concluded that the prevalence was similar in LAMICs and high-income countries (HICs), and that approximately one in three people experience a CMD at some point during their lifetime. Lifetime prevalence in LMICs was estimated to be lower than that in HICs [9]. However, there are some influential limitations in this review that warrant consideration in interpreting these conclusions. First, there was no critical engagement with whether the data collection tools (including diagnostic interviews) had been appropriately culturally verified or formally validated for the cultural contexts in which they were administered [10]. Second, there was also no consideration of cultural differences in emotional literacy or the impact on disclosure of severe stigma against people with mental disorders. It was not clear whether strategies to enable the participation of people with low literacy had been considered in evaluating study methodologies. If questions are not comprehensible people are likely to answer no and this leads to underestimates of prevalence.

Establishment of the prevalence of severe mental disorders in resource-constrained settings has been difficult due to insufficient mental health specialists and lack of research capacity in psychiatric epidemiology. The prevalence has most commonly been established by ascertainment of psychotic symptoms rather than diagnoses of disorders [11, 12]. In 2012 Nuevo et al. analysed data contributed by 256, 445 participants in 52 countries to the World Health Survey to establish the prevalence of psychotic symptoms. The cross-national survey employed the Composite International Diagnostic Interview (CIDI) administered trained by non-mental health professionals to detect specific psychotic symptoms, but not to form clinical diagnoses. The prevalence of having at least one psychotic symptom varied widely from 0.66 to 45.84%. The authors suggested that the potential reason for this wide range was cultural differences among countries. However, the publication did not mention the procedure of adapting the CIDI for specific countries. In addition to the cultural difference, the lack of formal validation might contribute to the wide variation of the prevalence of psychotic symptoms in the multi-national survey [13].

### Burden of mental disorders in Vietnam

There have been five investigations reported to date of the burden of CMDs and SMDs among adults in the general population in Vietnam in international peer-reviewed journals in English. Among these, one summarised the main findings of a Ministry of Health report, but did not include any information about method, sample and data analysis [14]. Four papers described four original research studies. One survey was conducted in a Southern province (Hau Giang) applying a multistage probabilistic cluster sampling frame with the commune or hamlet as the sampling unit. The investigators conducted interviews with 3039 people who were older than 17 years. Data collectors were mental health physicians, mental health nurses and general staff. The CIDI was culturally adapted in several stages including a comprehensive review of emotional states in Vietnamese ethnographic studies, and psychometric tests with high internal consistency (*r* = 0.87–0.95) and reliability (*r* = 0.81–0.89). The data collection tool consisted of a depression scale (26 items), an anxiety scale (13 items), and a somatisation scale (14 items) to measure the prevalence of depression and anxiety [15]. The household cross-sectional survey in two southern provinces (Da Nang and Khanh Hoa) used a four-stage cluster sampling strategy. The investigators included 4981 adults who were 18 or older. The face-to-face interviews were implemented by trained lay data collectors. The SRQ20 validated in Vietnam with the cut-off of 6/7 was used to screen for CMDs among the study population [16]. Another paper in Vietnam was a pre- and post Xangsane typhoon survey with 797 adults (18+) in Da Nang – a southern province. Data were collected by trained interviewers using validated SRQ20 with cut-off of 7/8 for CMDs [17]. Finally, the WHO survey was conducted in 52 countries including Vietnam. The CIDI was used by trained staff to screen for SMDs among 4174 adults (18+). This data collection tool was translated into Vietnamese using a specified protocol. There was lack of information about which province the study was conducted in [13].

The Ministry of Health reported that the prevalence of depression and anxiety among the population were 2.8 and 2.6% respectively [14]. The two peer-review articles revealed a wide variation of depression and anxiety prevalence. A survey among adults in the Southern Hau Giang province found that the prevalence was 1.2% (Standard Error: 0.7) for depression, 4.0% (Standard Error: 0.2) for anxiety [15]. The study in Da Nang and Khanh Hoa provinces reported that around 20% of adults have clinically significant symptoms of CMDs [16]. The prevalence in another study in Da Nang was 20.7% before the typhoon [17]. These findings suggest that the prevalence of CMDs among adults in Vietnam was estimated by screening instruments to be higher than among those using diagnostic tools.

The Ministry of Health reported the prevalence of schizophrenia to be 0.5% but means of ascertaining this was not clear [14]. The WHO survey found that the prevalence of having at least one psychotic symptoms in Vietnam was 0.66% (Standard Error: 0.20) at the population after weighted and sex-age standardization [13].

In general, the burden of CMDs and SMDs in Vietnam were estimated from sub-populations such as some provinces in the south of Vietnam, or from women during the perinatal period in the northern rural area. Methodologies used were inconsistent among these studies.

The four original studies in Vietnam reported a limited set of protective and risk factors such as age, gender, marital status, education, occupation [13–16]. In general, especially in rural areas, most families live in multi-generation arrangements where family members are strongly connected, share social capital, economic status, supports from government or resource groups, and co-experience adverse events. In addition, most people with psychotic disorders are cared by their family members. Therefore, mental health problems have to be understood within the family environment.

In order to address the policy need and limitation of the existing evidence in Vietnam, the aim was to estimate the burden of CMDs and SMDs among adults in Vietnam. In particular, this study aimed to (1) estimate the prevalence of clinically significant symptoms of CMDs and SMDs among adults in Vietnam; and (2) identify factors at both individual and family levels associated with CMDs and SMDs among adults in Vietnam.

## Methods

### Study design

The study was a cross-sectional household survey completed in May and June 2013.

### Setting

Vietnam is a World Bank classified lower-middle income country, with a population of more than 90 million people in Southeast Asia [18]. The North and the South of Vietnam have different socio-political characteristics. Historically, the two areas were ruled by different Kings with different cultures. More recently, the South area was influenced first by French colonisers and then the United States, whereas the North of Vietnam was influenced more by the Soviet Union and China. There remain significant differences between the two areas in terms of culture, idiomatic language, pronunciation, living standards, and social behaviours. The survey was conducted in two provinces which represent the north and the south of Vietnam. Thanh Hoa is the Northern province with the third highest population in Vietnam with 3.496.600 people living in 27 districts. The province has 6 coastal, 11 mountainous and 10 plain land districts and a capital city. At the time of the survey the average income per capita per month was approximately 1.6 million Vietnam dong (approximately USD 82) [19]. Ben Tre – a southern province has a population of approximately 1.26 million people living in one city and eight districts. All city and districts are in the plain land. The average income per capita per month was nearly 2.2 million Vietnam dong (more than USD 100) [20].

### Random selection of households and sample size

A cluster sampling method was used. At the provincial level, 30 communes were randomly selected from the list of communes provided by the provincial level Departments of Health and Labour, Invalids and Social Affairs. In each selected commune, a list of households was made by the commune administrators and 15 households were selected randomly from it. Ten households were visited and, if no-one was at home, a household was selected from the replacement list. All adults who were older than 16 years in the households were invited to participate in the survey. Household members were defined as members who have had meals together for at least 6 months. The household head was defined by the local authority. Commonly the household head is the most powerful decision-maker in the family.

### Data collection tools and sources

The structured interview schedules included standardised instruments and study-specific questions. Two forms of data were collected: (1) Individual-level information was collected by self-reported questionnaires. The questionnaires included the SRQ and other background information such as age, education, and gender; (2) Household socio-economic characteristics were ascertained by interviewing the head of the household.

Specific tools and information used in this survey were described in Table 1.

**Table 1:** Tools used for data collection

Variable	Tool	Description
Individual level		
Symptoms of common and severe mental disorders	WHO Self Reporting Questionnaire (SRQ)	The screening tool consists of 20 questions related to common non-psychotic mental disorders and four questions related to psychotic symptoms [12]. There are two formal validity studies of SRQ 20 items in Vietnam. The first study (2004) was conducted in a Northern province among 2000 female caregivers to validate the SRQ20 against the diagnosis of a professor of paediatric psychiatry. The cut-off point was 7/8 [21]. The second study in 2006 included a community sample of 500 people aged from 18 to 60 in a rural northern province presented an optimal cut-off point of 5/6. SRQ 20 items was validated against psychiatrists [22]. This study used the cut-off point of 6/7 to calculate the prevalence of clinically significant symptoms of CMDs among adults in Vietnam due to the similarity of the study participants. The SRQ includes 4 items assessing symptoms of severe mental disorders including (1) has somebody been trying to harm you in some way, (2) are you a much more important person than most people think, (3) have you noticed any interference or anything else unusual with your thinking, (4) have you ever heard voices without knowing where they come from or which other people cannot hear [12].
Household level		
Household economic status	One question addressing household economic status	Subjective self-assessment of household heads in terms of their household economic status compared to other neighbours in their commune [23].
Alcohol abuse	One question addressing whether any household member abused alcohol	The household heads identify whether a household member was abusing alcohol.
Family violence	Short form of domestic violence screening tool (HITS) [24].	The original four-item scale was developed to identify domestic violence in term of Hurts, Insults, Threatens and Screams (HITS) with 5-point frequency format at individual level. The score ranges from 4 (no family violence) to 20 (frequent family violence) [24]. This scale was adapted to screen the family violence by asking whether any household member suffering from such domestic violence.
Social capital	Short version of the modified Adapted Social Capital Assessment Tool (SASCAT) [25]	The SASCAT comprises nine items divided into two subscales: structural and cognitive social capital. The structural social capital reflects the connectedness of the household with the community such as participation in organizations, degree of citizenship (the involvement in voting, campaign activities at commune or provincial levels), specific collective action (together with other neighbour to address a specific problem in the community) and links to groups with resources (such as local government or aid agencies). The cognitive social capital describes the feelings of sense of community such as reciprocity, and sharing. It consists of emotional support, instrumental support (food, money, etc), trust, social harmony (getting along with others in the community), sense of belonging (attachment to the community), and perceived fairness (whether others in the community take advantage of people) [26]. This tool was validated in Vietnam with nearly 80% of accurate interpretation of all questions among caregivers and data collectors. SASCAT was also reported as a valid tool in terms of psychometric and cognitive validation [27, 28].
Specific adverse life event	One question assessing experiences of adverse life events	Have the following events occurred in the previous 3 years: household items stolen/robbed, threatening of losing or lost rights of inheritance, threatening or lost land/property ownership.

### Procedure

Local staff of the provincial level Department of Health and Department of Invalids and Social Affairs were employed as data collectors due to their familiarity with local accents, customs, and access routes to communes. They were trained for 3 days by the research team from the Research and Training Centre for Community Development (RTCCD) in Hanoi. Only staff who fulfilled all requirements in terms of communication skills and understanding of the questionnaire, protection of confidentiality and ensuring data integrity were employed as data collectors.

All potential participants were informed by local authorities that they were to be invited for interviews. At the day of the interview, village heads guided the data collectors to the selected households. All household members who were adults were given separately an oral or written plain language description of the study and were asked to sign a consent form. Those who could not write provided a thumbprint or verbal consent witnessed by an independent observer. After interviewing the household heads to collect household characteristics, all members who were older than 16 years old were assigned into different private rooms to complete the written self- report the SRQ with 20 items for symptoms of CMDs and 4 items for psychotic symptoms. For those who had low literacy, interviews were conducted by the data collectors.

The research team supervised all data collectors and selected randomly 5% of the completed questionnaires to reinterview participants for quality assurance. In addition, questionnaires were checked by supervisors in the field for missing values and inconsistent responses, to enable data collectors to go back to the household to amend possible errors.

Completed consent forms and paper-based questionnaires gathered by the data collectors were returned to the research team at the end of each working day for secure storage in a locked cabinet at the provincial level Department of Labour, Invalids and Social Affairs office. Then it was transported in a locked bag with the research team for secure storage and data entry at the RTCCD office.

### Data management and analysis

No name was recorded in the questionnaires, only the codes of provinces, communes and households were used for data entry and data analysis. Family members shared the same family code, but different personal codes to allow the researchers to identify data of individual and household levels.

A double data entry method was conducted using the Microsoft Access 2013 (Microsoft Corp, USA). The paper records were stored securely at RTCCD office and only researchers had access to the original data.

Data analyses were completed in two steps using Stata, Version 13.0 (StataCorp LP, College Station, Texas, USA), with statistical significance set at *p*-value less than 0.05. Step 1 used descriptive analyses to calculate the community prevalence and 95% confidence intervals. A score of 6 and above on the SRQ20 was used as the indicator of clinically-significant symptoms of CMDs. The cut-off score of 5/6 was reported in the validity study of SRQ-20 with a sensitivity of 85%, and a specificity of 46% [22]. Participants who reported at least one psychotic symptom were classified as having a clinically significant symptom of an SMD [12]. People who reported both clinically significant symptoms of CMDs and any psychotic symptoms were classified in the SMD group [29]. The prevalence of CMDs and SMDs were weighted using the standard proportion of age and sex groups among the Vietnam population in 2013 [30].

Step 2, univariate analyses were used to compare individual characteristics between groups of people with and without clinically-significant symptoms of CMDs or SMDs. The same method was also employed to identify differences in household characteristics between those with or without member having clinically significant symptoms of CMDs or any symptom of SMDs.

In step 3, the binary outcome (CMDs or SMDs) a value of 0 was given to the category of having no clinically significant symptoms of CMDs or SMDs, and a value of 1 to the category of having clinically significant symptoms. The data of this study had two levels, with individuals nested within households. All people in the same household shared common socio-economic characteristics. Therefore, two-level mixed-effects logistic regressions with random intercepts and fixed coefficients including both household and individual characteristics were used [31].

## Results

In total 643 households were approached, among which 32 households were not included either because the household heads were not at home at the time the study team visited or declined participation. In total, 611 households accepted the invitation to contribute data, giving a recruitment fraction of 95%. Overall, 1528 individual household members aged more than 16 years contributed data.

### Socio-economic characteristics of the study sample

Most households were of mid-level or disadvantaged economic status (72.6%) and had experienced specific adverse events in the previous 3 years. Regarding the cognitive social capital, a majority of households reported experiencing emotional support, trust, social harmony, and sense of belonging. Insignificant numbers reported any experiences of family violence. Among 1528 participants, most were aged over 24 years and less than 10% had completed tertiary education. More women than men contributed data (see Table 2).

**Table 2:** Social characteristics of households and individuals in Vietnam which contributed data

Variables	Thanh Hoa n (%)	Ben Tre n (%)	Total N (%)
Household level			
Number of households	294 (48.1)	317 (51.9)	611 (100%)
Economic status			
Disadvantaged	51 (17.3)	50 (15.8)	101 (16.5)
Mid-level	181 (61.6)	174 (54.9)	355 (58.1)
Advantaged	62 (21.1)	93 (29.3)	155 (25.4)
Residence			
Urban	50 (17.0)	169 (53.3)	219 (35.8)
Rural	244 (83.0)	148 (46.7)	392 (64.2)
Alcohol abuse in the family			
Yes	23 (7.8)	52 (16.4)	75 (12.3)
No	271 (92.2)	265 (83.6)	536 (87.7)
Family violence			
Yes	9 (3.1)	5 (1.6)	14 (2.3)
No	285 (96.9)	312 (98.4)	597 (97.7)
Structural social capital			
Participation in organizations	72 (24.5)	145 (45.7)	217 (35.5)
Degree of citizenship	204 (69.4)	227 (71.6)	431 (70.5)
Specific collective actions	90 (30.6)	136 (42.9)	226 (37.0)
Links to groups with resources	92 (31.3)	196 (61.8)	288 (47.1)
Cognitive social capital			
Emotional support	263 (89.5)	226 (71.3)	489 (80.0)
Instrumental support	203 (69.1)	180 (56.8)	383 (62.7)
Trust	271 (92.2)	269 (84.9)	540 (88.4)
Social harmony	286 (97.3)	301 (95.0)	587 (96.1)
Sense of belonging	289 (98.3)	303 (95.6)	592 (96.9)
Perceived fairness	275 (93.5)	301 (95.0)	576 (94.3)
Specific adverse events within the last 3 years			
Yes	240 (81.6)	248 (78.2)	488 (79.9)
Household size (Mean ± SD)	3.5 ± 1.4	3.5 ± 1.4	3.5 ± 1.4
Individual level			
Number of participants	646 (42.3)	882 (57.7)	1528 (100%)
Age			
17–24 years old	80 (12.4)	108 (12.2)	188 (12.3)
25–49 years old	251 (38.8)	434 (49.2)	685 (44.8)
50+ years old	315 (48.8)	340 (38.6)	655 (42.9)
Gender			
Male	288 (44.6)	389 (44.1)	677 (44.3)
Female	358 (55.4)	493 (55.9)	851 (55.7)
Education			
Up to completed primary school	159 (24.6)	464 (52.6)	623 (40.8)
Completed secondary school	439 (68.0)	334 (37.9)	773 (50.6)
Any post-secondary qualification	48 (7.4)	84 (9.5)	132 (8.6)

### The prevalence of clinically significant symptoms of CMDs and SMDs

Among the 1528 participants, 251 (16.4% (95% Confidence Interval: 14.65–18.37%)) had SRQ20 scores greater than 6/20, and 132 (8.6% (95% Confidence Interval: 7.32–10.16%)) reported at least one psychotic symptom. After weighting for age and sex, the population point prevalence of CMDs was 14.2% (95% Confidence Interval: 14.16% - 14.18), and the population lifetime prevalence of SMDs was 8.1% (95% Confidence Interval: 8.12–8.14%).

### Factors associated with CMDs

In the uncontrolled univariate analyses, individual characteristics including gender, age, and education were significantly associated with CMDs (see Table 3). The household factors which were significantly associated with CMDs were province, residence of urban or rural area, economic status, and some social capital items (specific collective actions, links to groups with resources, instrumental support, and social harmony) (see Table 4).

**Table 3:** Univariable comparison of characteristics of people with or without a SRQ20 score > 6

	People without clinically significant symptoms of CMDs n (%)	People with clinically significant symptoms CMDs n (%)	Total n (%)	*P*-value
Sex				
Male	588 (46.1)	89 (35.5)	677 (44.3)	0.002
Female	689 (53.9)	162 (64.5)	851 (55.7)	
Age				
17–24 years old	176 (13.8)	12 (4.8)	188 (12.3)	< 0.001
25–49 years old	610 (47.8)	75 (29.9)	685 (44.8)	
50+ years old	491 (38.4)	164 (65.3)	655 (42.9)	
Education				
Up to completion of primary school	502 (39.3)	121 (48.2)	623 (40.8)	< 0.001
Completion of secondary or high school	648 (50.7)	125 (49.8)	773 (50.6)	
TAFE/ bachelor/postgraduate	127 (10.0)	5 (2.0)	132 (8.6)

**Table 4:** Univariable comparison of characteristics of households with or without a member with SRQ20 scores > 6

	Household with no members having clinically significant symptoms of CMDs n (%)	Household with members having clinically significant symptoms of CMDs n (%)	Total n (%)	*P*-value
Province				
Thanh Hoa (north)	230 (45.9)	64 (58.2)	294 (48.1)	0.02
Ben Tre (south)	271 (54.1)	46 (41.8)	317 (51.9)	
Residence				
Urban	189 (37.7)	30 (27.3)	219 (35.8)	0.01
Rural	212 (62.3)	80 (72.7)	392 (64.2)	
Household size (Mean ± SD)	3.5 ± 1.4	3.3 ± 1.4	3.5 ± 1.4	0.08
Economic status				
Disadvantaged	73 (14.6)	28 (25.5)	101 (16.5)	0.03
Mid-level	296 (59.1)	59 (53.6)	355 (58.1)	
Advantaged	132 (26.3)	23 (20.9)	155 (25.4)	
Alcohol abuse	53 (10.6)	22 (20.0)	75 (12.3)	0.06
Family violence (Mean ± SD)	4.0 ± 0.2	4.0 ± 0.2	4.0 ± 0.2	0.3
Structural social capital				
Participation in organizations	185 (36.9)	32 (29.1)	217 (35.5)	0.1
Degree of citizenship	351 (70.1)	80 (72.7)	431 (70.5)	0.5
Specific collective actions	195 (38.9)	31 (28.2)	226 (37.0)	0.03
Links to groups with resources	246 (49.1)	42 (38.2)	288 (47.1)	0.003
Cognitive social capital				
Emotional support	398 (79.4)	91 (82.7)	489 (80.0)	0.4
Instrumental support	327 (65.3)	56 (50.9)	383 (62.7)	0.005
Trust	446 (89.0)	94 (85.5)	540 (88.4)	0.2
Social harmony	485 (96.8)	102 (92.7)	587 (96.1)	0.04
Sense of belonging	485 (96.8)	107 (97.3)	592 (96.9)	0.7
Perceived fairness	475 (94.8)	101 (91.8)	576 (94.3)	0.2
Adverse events within the last 3 years	99 (19.8)	24 (21.8)	123 (20.1)	0.6

After conducting the multilevel mixed-effects logistic regression, participants who were younger than 24 years old versus 50+ group, male and had completed tertiary education were less likely to experience clinically significant symptoms of common mental disorders. Households in the southern province, having advantaged economic status, having no member experiencing alcohol abuse, having links to groups with resources and receiving instrumental support were protected against CMDs among their family members (see Table 5).

**Table 5:** Adjusted odd ratios of household and individual characteristics of adults with and without SRQ20 scores> 6 in Vietnam

Factors	Adjusted OR	95% CI	*P*-value
Age			
0–17-24 years old (reference group)			
25–49 years old	1.69	0.83–3.43	0.1
50+ years old	4.64	2.27–9.47	< 0.001
Gender			
Male (reference group)			
Female	1.59	1.14–2.21	0.006
Education			
TAFE/bachelor/postgraduate (reference group)			
Completion of secondary or high school	3.73	1.35–10.38	0.01
Up to completion of primary school	4.57	1.61–13.0	0.004
Province			
Thanh Hoa (northern Vietnam) (reference group)			
Ben Tre (southern Vietnam)	0.48	0.30–0.75	0.002
Economic status			
Disadvantaged (reference group)			
Mid-level	0.62	0.38–1.01	0.06
Advantaged	0.53	0.29–0.98	0.04
Residence			
Urban (reference group)			
Rural	1.02	0.66–1.59	0.9
Household size	1.01	0.89–1.15	0.8
Alcohol abuse			
No (reference group)			
Yes	1.80	1.06–3.06	0.03
Family violence			
No (reference group)			
Yes	1.99	0.71–5.58	0.1
Structural social capital			
Participation in organizations			
Yes (reference group)			
No	1.08	0.70–1.65	0.7
Degree of citizenship			
Yes (reference group)			
No	1.02	0.67–1.57	0.9
Specific collective action			
Yes (reference group)			
No	0.77	0.50–1.19	0.2
Links to groups with resources			
Yes (reference group)			
No	1.83	1.16–2.88	0.009
Cognitive social capital			
Emotional support			
Yes (reference group)			
No	1.45	0.85–2.49	0.1
Instrumental support			
Yes (reference group)			
No	1.56	1.04–2.34	0.03
Trust of community			
Yes (reference group)			
No	1.23	0.65–2.33	0.5
Social harmony			
Yes (reference group)			
No	1.75	0.62–4.90	0.2
Sense of belonging			
Yes (reference group)			
No	1.01	0.32–3.22	0.9
Perceived fairness			
Yes (reference group)			
No	1.88	0.91–3.85	0.08
Adverse life events in the last 3 years			
No (reference group)			
Yes	1.00	0.64–1.58	0.9

### Associated factors of SMDs

In the uncontrolled univariate analyses, at individual level, gender, age, and education were not significantly associated with symptoms of SMDs (see Table 6). Household’s factors associated with any psychotic symptom were province, residence, and economic status (see Table 7).

**Table 6:** Univariable comparison of characteristics of people with or without any psychotic symptoms

	People without any psychotic symptoms n (%)	People with any psychotic symptoms n (%)	Total n (%)	*P*-value
Gender				
Male	628 (45.0)	49 (37.1)	677 (44.3)	0.08
Female	768 (55.0)	83 (62.9)	851 (55.7)	
Age				
17–24 years old	178 (12.8)	10 (7.6)	188 (12.3)	0.08
25–49 years old	630 (45.1)	55 (41.7)	685 (44.8)	
50+ years old	588 (42.1)	67 (50.8)	655 (42.9)	
Education				
Up to completion of primary school	576 (41.3)	47 (35.6)	623 (40.8)	0.4
Completion of secondary or high school	701 (50.2)	72 (54.6)	773 (50.6)	
TAFE/ bachelor/postgraduate	119 (8.5)	13 (9.8)	132 (8.6)

**Table 7:** Univariable comparison of characteristics of households with or without a member with at least one psychotic symptom

	Household with no members having any symptom of SMDs n (%)	Household with members having any symptom of SMDs n (%)	Total n (%)	*P*-value
Province				
Thanh Hoa (north)	260 (46.9)	34 (60.7)	294 (48.1)	0.04
Ben Tre (south)	295 (53.1)	22 (39.3)	317 (51.9)	
Residence				
Urban	207 (37.3)	12 (21.4)	219 (35.8)	0.01
Rural	348 (62.7)	44 (78.6)	392 (64.2)	
Household size (Mean ± SD)	3.5 ± 1.4	3.3 ± 1.4	3.5 ± 1.4	0.08
Economic status				
Disadvantaged	83 (14.9)	18 (32.1)	101 (16.5)	0.002
Mid-level	325 (58.6)	30 (53.6)	355 (58.1)	
Advantaged	147 (26.5)	8 (14.3)	155 (25.4)	
Alcohol abuse	71 (12.8)	4 (7.1)	75 (12.3)	0.2
Family violence (Mean ± SD)	4.0 ± 0.2	4.1 ± 0.04	4.0 ± 0.2	0.1
Structural social capital				
Participation in organizations	200 (36.0)	17 (30.4)	217 (35.5)	0.3
Degree of citizenship	393 (70.8)	38 (67.9)	431 (70.5)	0.5
Specific collective action	204 (36.8)	22 (39.3)	226 (37.0)	0.7
Links to groups with resources	267 (48.1)	21 (37.5)	288 (47.1)	0.1
Cognitive social capital				
Emotional support	442 (79.6)	47 (83.9)	489 (80.0)	0.4
Instrumental support	354 (63.8)	29 (51.8)	383 (62.7)	0.07
Trust	489 (88.1)	51 (91.1)	540 (88.4)	0.5
Social harmony	533 (96.0)	54 (96.4)	587 (96.1)	0.8
Sense of belonging	538 (96.9)	54 (96.4)	592 (96.9)	0.8
Perceived fairness	522 (94.1)	54 (96.4)	576 (94.3)	0.4
Adverse events within the last 3 years	113 (20.4)	10 (17.9)	123 (20.1)	0.6

The multilevel mixed-effects logistic regression analysis revealed that, people aged at least 50 years were more likely to report symptoms of SMDs than those in the 17–24 years old group. Households in the southern province, having mid-level or advantaged economic status and having no family violence were less likely to have family members with any psychotic symptom (see Table 8).

**Table 8:** Adjusted odd ratios of household and individual characteristics of adults with and without any psychotic symptom 6 in Vietnam

Factors	Adjusted OR	95% CI	*P*-value
Age			
17–24 years old (reference group)			
25–49 years old	2.15	0.98–4.71	0.06
50+ years old	2.83	1.26–6.38	0.01
Gender			
Male (reference group)			
Female	1.42	0.94–2.14	0.09
Education			
TAFE/bachelor/postgraduate (reference group)			
Completion of secondary or high school	0.69	0.32–1.49	0.3
Up to completion of primary school	0.47	0.20–1.09	0.07
Province			
Thanh Hoa (northern Vietnam) (reference group)			
Ben Tre (southern Vietnam)	0.54	0.31–0.95	0.03
Economic status			
Disadvantaged (reference group)			
Mid-level	0.50	0.27–0.91	0.02
Advantaged	0.35	0.16–0.75	0.007
Residence			
Urban (reference group)			
Rural	1.25	0.72–2.16	0.4
Household size	1.00	0.85–1.18	0.9
Alcohol abuse			
No (reference group)			
Yes	0.58	0.26–1.30	0.1
Family violence			
No (reference group)			
Yes	3.50	1.08–11.31	0.03
Structural social capital			
Participation in organizations			
Yes (reference group)			
No	1.02	0.60–1.72	0.9
Degree of citizenship			
Yes (reference group)			
No	1.00	0.57–1.71	0.9
Specific collective action			
Yes (reference group)			
No	1.12	0.65–1.92	0.6
Links to groups with resources			
Yes (reference group)			
No	1.01	0.58–1.75	0.9
Cognitive social capital			
Emotional support			
Yes (reference group)			
No	1.27	0.65–2.49	0.4
Instrumental support			
Yes (reference group)			
No	1.54	0.93–2.56	0.09
Trust of community			
Yes (reference group)			
No	1.37	0.63–2.98	0.4
Social harmony			
Yes (reference group)			
No	1.10	0.28–4.35	0.8
Sense of belonging			
Yes (reference group)			
No	0.87	0.18–4.24	0.8
Perceived fairness			
Yes (reference group)			
No	0.55	0.18–1.65	0.2
Adverse life events in the last 3 years			
No (reference group)			
Yes	1.06	0.60–1.87	0.8

## Discussion

To our knowledge, this is the first household survey conducted in both southern and northern provinces to estimate the burden of common and severe mental disorders among adults in Vietnam. A rigorous random selection method was used in each implementation step and there was a high recruitment fraction (95%). The data were collected by local language speakers to maximize the likelihood that participants could comprehend the questions. The data sources were developed in Vietnam and the indicator of the primary outcome of the SRQ20 had been formally validated to establish the local cut-off scores for clinically significant symptoms with high sensitivity and specificity in the country.

In rural provinces in Vietnam, it is common for people to migrate internally to secure income-generating work. Elderly and young people are more likely to stay at home to farm while people of working age migrate to urban centres for paid employment. In order to take this into account, prevalence estimates were weighted by age group according to the national household survey conducted in the same year. No previous comparable studies in Vietnam have made this adjustment.

Nevertheless, we acknowledge some limitations in the study.

Relevant information about individuals was not ascertained directly, but was sought from household heads. This practice, while common in household surveys in LMICs introduces potential biases, which might reduce precision of prevalence estimates and accuracy of associations with risk and protective factors. There is a likely gender bias in that most household heads are men and might have limited understanding or appreciation of the experiences of women. Additionally, household heads may have been unlikely to disclose their own perpetration of family violence or alcohol abuse. These risks are therefore most likely to be underestimated.

Alcohol abuse was not fully investigated. It was addressed by asking one question to the household heads to ascertain whether any family member was misusing alcohol. The definition of alcohol abuse was likely to have varied among household heads and was not defined clearly by the research team [15].

Finally, SRQ is a psychometric tool which was used to identify probable cases of clinically significant symptoms of CMDs and SMDs in community [12]. In other words, this tool indicates that it cannot provide a diagnosis as a diagnostic instrument. Therefore, SRQ results can only suggest potential burden of mental disorders in a population.

Despite these limitations which we believe are more likely to have led to under rather than overestimates of prevalence, we believe the data provide a generalizable indication of the burden and potentially modifiable risks for mental disorders in Vietnam.

### Prevalence of clinically significant symptoms of CMDs and SMDs in Vietnam and other countries

Overall, the prevalence of clinically significant symptoms of CMDs and SMDs among people who are aged at least 16 years old in this study was more than 22% after age standardization. The raw prevalence of clinically significant symptoms of CMDs was around 20%, which is similar to other studies using SRQ20 [16, 17]. However, there were people who had both clinically significant symptoms of CMDS and SMDs which was included in the group of having CMDs. The DSM-5 reported that people with psychotic disorders may sometimes experience several depressive and anxiety periods during their lifetime [29]. Therefore, after excluding those people in the CMDs’ group, the prevalence reduced from around 20% to just over 14%.

The rate of clinically significant symptoms of CMDs is lower than other studies using SRQ20 among adults in southern provinces [16, 17]. There are several factors contributing to the difference. First, it is due to the deduction of people having both CMDs and SMDs symptoms. Second, the difference may be due to the different cut-off point (7/8) and lack of age standardization. However, the result of this study is higher compared to other three studies [14, 15, 32]. The study conducted by Kim Bao Giang et al. reported a low prevalence (5.4%) using the same screening tool (SRQ20). Differences in age distribution and geographical area may be the possible explanations. Giang et al’s study was implemented among adults from 18 to 60 years old and was not weighted with the standard age distribution of Vietnam. In addition, Ba Vi is a northern district of Hanoi city, which is around 60 km from the centre of Hanoi. Therefore, the socio-economic status may be more advantaged compared to our study population which may lead to the lower rate of CMDs than our study [32]. Different psychometric instruments used across studies for identifying the burden of mental health problems are also considered as a reason of the difference. The study conducted by Steel et al. in a southern province use CIDI – a diagnostic tool with cultural adaptation [15]. The rate of depression and anxiety reported in the Vuong et al. paper did not provide sufficient details for discussion of the difference [14].

Our finding of 8.6% of SMDs was higher than the result in Vietnam (0.66%), but lower than the prevalence of low and middle income countries (12.9%) which were reported by a WHO multi-country study using CIDI [13]. The rate of respondents having one or more psychotic symptoms in this survey is higher than the prevalence of schizophrenia published by the Ministry of Health (0.5%) [14]. The World Health Survey used CIDI – a diagnostic tool and the Vietnam government’s prevalence is believed to employ diagnostic instruments because it was estimated by the public mental health hospitals which may explain the different findings. However, due to the limitation to access to full report of the Ministry of Health’s report, therefore, it is hard to provide further evidence for the difference.

### Determinants of CMDs among different demographic groups

Regarding individual factors, there was a larger proportion of people with CMDs among women than man which was consistent with other studies [32, 33]. It may due to gender inequity which creates a significant burden on women in Vietnam. In this study, people who are older than 50 years old faced a significant higher risk of both CMDs and SMDs compared to the 15–24 years old while others presented that CMDs were even less than in elderly or no association [6, 32, 34, 35]. It can be explained that, in rural Vietnam, most study participants were farmers, therefore the older they were, the less probability to have a secure income from their farming. As a result, they were more likely to experience financial difficulties compared to younger participants. Illiteracy was found to be a risk factor for CMDs [32, 36]. The reason may be that there is a strong link between education level and employment status. People with low literacy are less likely to get a job, they may have lower income. Therefore, less educated people had higher risk of experience CMDs.

In terms of determinants at the family level, living in Ben Tre (the southern province), and in a more economically advantaged household were found to independently protect adults from SMDs and CMDs. It can be seen that Ben Tre province is wealthier and has more natural resources compared to Thanh Hoa – a poor province in the north of Vietnam. Therefore, the hard-living condition in the north province contributed to the higher prevalence of both SMDs and CMDs. Similarly, households having more advantaged economic status compared to the disadvantaged group were less likely to have family members experience CMDs and SMDs. This result was confirmed by other studies in LMICs [32, 37, 38]. It is also well known that there was a strong link between poverty and CMDs in LMICs by Lund and et al. [36]. Another household factor found to be associated with CMDs was there being at least one member experiencing alcohol abuse in the family. It may be that having at least one family member misusing alcohol increases the risk of both physical and emotional violence, and poverty. These risks, in turns, affect to the mental health of family members negatively [16, 39–42]. However, due to the acknowledged limitation in examining the family violence in this study, hence, there was no association between CMDs and this factor.

It is believed that emotional support tends to be the most effective method when someone is experiencing depressive or anxiety symptoms [43]. However, this analysis did not find this association. The data showed that having no instrumental support or no link to groups with resources was associated with CMDs. In this study, households having disadvantaged economic status were more likely to have family members experiencing CMDs. This may suggest that material support (for example: money, food) meets the basic needs of these households and alleviates their stress. In return, it may improve their mental health.

### Determinants SMDs among different demographic groups

At individual level, we can only speculate on the reason for their being a higher prevalence of SMD symptoms among older than younger people. It may due to the three underlying reasons. First, it was reported in a recent population-based survey that among current drinkers, those aged around 50+ years old consumed the most alcohol [44]. Alcohol abuse is believed to be associated with psychosis [29]. Second, there are no community-based mental health services to provide assessment, diagnosis or treatment for people with psychotic symptoms in rural Vietnam. This service is only provided in custodial psychiatric hospitals. These are stigmatised institutions, about which people generally feel cautious. In addition, many people in rural areas have disadvantaged economic circumstances and cannot afford the travel costs to go to psychiatric hospitals [45]. These might lead to prolonged delays to diagnosis and treatment among people in the 50+ age group. Third, another reason may be the late – onset psychosis. A recent study showed that there is not a small number of cases having psychotic symptoms in the late life [46]. In addition, there was a strong link between people who were up to completion of primary school and psychotic symptoms. Substantial evidence of the association between school performance and psychotic disorders was established [47, 48]. Early cognitive alteration was believed to hinder the education achievement.

At household level, similar to the associating factors of CMDs, residence and economic status had significant link to SMDs. At the time that the survey was conducted, the average income per capital per month in the Southern province was higher than that in the Northern province. In addition, the relationship between economic disadvantage and psychotic symptoms was well established in previous research studies [49, 50]. People having psychotic symptoms are less likely to have stable incomes and more likely to live in poverty due to their low productivity [51], limited educational level [52]. Family violence was found as a risk factor of having psychotic symptoms in this study. On the basis of this data, it is not possible to identify who is the perpetrator of the family violence. People with SMDs might be the victim, or the perpetrator, or the witness of the family violence. A systematic review of 41 studies conducted by Trevillion et al. in 2012 found that people with SMDs were at risk of experiencing domestic violence [53]. However, included studies were reported to have limited quality in terms of selection bias, sample representativeness, and impact of non-participation. In addition, domestic violence was measured using various methods such as different time periods, types of violence, and instruments. On the other hand, a study mentioned the burden of violent behaviour among caregivers of people with schizophrenia and bipolar disorders. With the sample of 243 schizophrenic patients and 200 bipolar patients recruited within 1 week of patient’s admission to the mental health hospital, Zhou et al. found that caregivers of people with bipolar disorders experienced more violent burden than those of schizophrenic people [54]. The findings were reported among recent admission patients, hence, the caregivers may experience significant burden than the people with SMI in the community due to the patients’ acute symptoms. In addition, due to the domestic violence, people were more likely to admit the question of “somebody has been trying to harm you in some way”.

## Conclusions

The study found that among people who are older than 16 years old, around 14% of them experienced CMDs and approximately 8% had symptoms of SMDs. This burden indicates the need for early detection at primary health care. In addition, except for gender and age, most common influencing factors related to family level. The result suggests that instead of focusing on the individual level, the community-based mental health model in Decision 1215 should focus on reducing family hazards such as family violence and providing instrumental support and access to resource groups to those in need.

## Data Availability

The datasets used and/or analysed during the current study are available from the corresponding author upon reasonable request.

## Abbreviations

CIDI: Composite International Diagnostic Interview
CMD: Common mental disorders
HICs: High income countries
HITS: Hurts, Insults, Threatens and Screams
LMICs: Low and middle-income countries
RTCCD: Research and Training Centre for Community Development
SASCAT: Short version of the modified Adapted Social Capital Assessment Tool
SMD: Severe mental disorders
SRQ: Self-reporting Questionnaire
WHO: World Health Organization
YLD: Years lived with disability
